# Functional spectroscopic imaging reveals specificity of glutamate response in mouse brain to peripheral sensory stimulation

**DOI:** 10.1038/s41598-019-46477-1

**Published:** 2019-07-22

**Authors:** Aline Seuwen, Aileen Schroeter, Joanes Grandjean, Felix Schlegel, Markus Rudin

**Affiliations:** 1 Institute for Biomedical Engineering, University and ETH Zurich, Wolfgang-Pauli-Str. 27, 8093 Zurich, Switzerland; 2 Neuroscience Center Zurich, University and ETH Zurich, Winterthurer-Str. 190, 8057 Zurich, Switzerland; 3 Singapore BioImaging Consortium, Agency for Science, Technology, and Research, 11 Biopolis way, Singapore, 138667 Singapore; 4 Institute of Pharmacology and Toxicology, University of Zurich, Winterthurer-Str. 190, 8057 Zurich, Switzerland

**Keywords:** Sensory processing, Translational research

## Abstract

Non-invasive investigation of physiological changes and metabolic events associated with brain activity in mice constitutes a major challenge. Conventionally, fMRI assesses neuronal activity by evaluating activity-evoked local changes in blood oxygenation levels (BOLD). In isoflurane-anaethetized mice, however, we found that BOLD signal changes during paw stimulation appear to be dominated by arousal responses even when using innocuous stimuli. Widespread responses involving both hemispheres have been observed in response to unilateral stimulation. MRS allows probing metabolic changes associated with neuronal activation and provides a complementary readout to BOLD fMRI for investigating brain activity. In this study we evaluated the sensitivity of a free induction decay (FID) based spectroscopic imaging (MRSI) protocol for the measurement of alterations in glutamate levels elicited by unilateral electrical paw stimulation at different current amplitudes. Coronal MRSI maps of glutamate distribution with 17 × 17 voxels of 1 µl volume have been recorded with a temporal resolution of 12 min. Significant region-specific increases in glutamate levels have been observed in the contralateral but not in the ispiateral S1 somatosensory cortex upon stimulation. The amplitude of glutamate changes increased in a dose-dependent manner with the stimulus amplitude. The study demonstrates feasibility of functional MRSI in mice for studying activity-evoked glutamate changes in a temporo-spatially resolved manner.

## Introduction

Neural activity elicits a sequel of physiological and metabolic changes to account for the increased energy/oxygen demand by tissue. Alterations in the balance of oxygen extraction to oxygen delivery induce changes in blood flow and blood oxygenation, which are assessed by conventional functional magnetic resonance imaging (fMRI). The hemodynamic nature of the readout renders it susceptible to confounds caused by systemic alterations in physiology. In particular in mice conflicting results have been reported when using paw stimulation paradigms^1–4^. We found that in isoflurane-anesthetized mice quantification of changes in the BOLD fMRI signals elicited by sensory (e.g. electrical) paw stimulation was confounded by stimulus-induced alterations in cardiovascular parameters potentially associated with arousal^4–6^. Such influences of systemic physiology on cerebral hemodynamic response may mask specific stimulus-elicited fMRI signals. Strategies to minimize the contribution from systemic changes in hemodynamic parameters might include modified stimulation protocols^7^ but also the use of non-vascular activity readouts.

Functional proton magnetic resonance spectroscopy (^1^H-MRS) measuring signals arising from endogenous metabolites and neurotransmitters in a time-resolved manner constitutes an alternative readout of brain function, which is not based on cerebral hemodynamics and therefore should be less susceptible to systemic confounds. Of the various metabolite signals identified in the MR spectrum, several are directly associated with neurotransmission (glutamate, Glu; glutamine, Gln; gamma-aminobutyric acid, GABA) or energy turnover (lactate, Lac; glutamate, Glu; phosphocreatine, PCr; creatine, Cr). Quantitative assessment of changes of metabolite signal intensities, in particular that of the major excitatory neurotransmitter Glu, has been suggested as indicator of neural activity^8–11^. In humans and rats, single voxel MR spectroscopy (SVS) was successfully applied to characterize neurotransmitter and metabolic changes during visual^9,12–15^, motor and sensory^16–18^ stimulation. Common observations include changes related to the increased energy demand of active brain areas, as reflected by elevated concentrations of Lac indicative of glycolytic energy production, and a decreased ratio phosphocreatine/creatine (PCr/Cr) reflecting replenishing of ATP levels via the creatine kinase reaction^19,20^. Yet, the most robust changes across recent studies performed at high field strength relate to altered levels of the neurotransmitter Glu, which were reported increased by 2 to 4% in humans depending on stimulus strength and type^8–10,18,21,22^. In anesthetized rats, increased in Glu levels upon electrical stimulation of the trigeminal nerve reached 8%^16^. Up to date, technical hurdles related to the high demands on spatial resolution and correspondingly on intrinsic SNR have hindered similar measurements in mice. The recording of a SVS data set for voxel volumes compatible with the size of mouse brain areas involved in sensory processing typically exceeds 30 min, limiting its use for dynamic measurements. Moreover, SVS does not allow for direct comparative analysis of metabolic alterations across brain areas, which would be relevant for assessing differential responses to peripheral input. Particularly in mice, in which we found the BOLD response to appear widespread and unspecific, a direct comparison between activated and control regions is crucial in order to access the specificity of the metabolic response.

Previously, we proposed a ^1^H-MRS approach based on spectroscopic imaging (SI) for mapping metabolite and neurotransmitter distribution in the mouse brain at 1 µl nominal spatial and 12 min temporal resolution^23^. MRSI allows covering significant brain area, thereby enabling the comparison of stimuli-induced changes in metabolite profiles in responsive and non-responsive brain areas. On the other hand, MRSI generally bares the risk of inferior shim quality across the area of interest, and of contamination of spectra by lipid signals, in particular for voxels close to the brain surface. The MRSI approach proposed is based on the acquisition of the free induction decay (FID) signal and thus provides increased signal intensity and minimal chemical shift displacement artifacts^24^ as compared to spin-echo based data acquisition, and therefore constitutes an attractive alternative to SVS for functional measurements in mice. We have used the method earlier to quantify dose-dependent changes in levels of neurotransmitters and metabolites involved in energy metabolism in response to GABA_A_ receptor inhibition in a region-dependent manner^23^. In the present study, we have applied the MRSI approach to assess regional Glu changes in response to sensory input, i.e. electrical stimulation of the mouse hindpaw. Unilateral paw stimulation led to significant increases in Glu signal intensity in the corresponding contralateral but not in the ipsilateral somatosensory cortical region. Glu signal changes in the contralateral somatosensory cortex depended on the stimulus strength. In contrast, BOLD fMRI responses were of widespread bilateral nature even though they were found to correlate with the stimulus amplitude. It appears that activity measures based on neurotransmitter levels are less susceptible to systemic confounds than those based on neuro-vascular hemodynamic coupling and thus might reflect neural activity more intimately, at least when studying sensory stimulation in mice.

## Materials and Methods

### Animal preparation

All *in vivo* experiments comply with Swiss Animal Protection Act and Ordinance. Animal experiments were approved by the Veterinary Office of Canton Zürich, upon the recommendation of Cantonal Commission for Animal Experiments Zürich, Switzerland, and are compliant with the ARRIVE guidelines (license number ZH242/17). Animal preparation followed the procedure described in^23^. Female C57BL/6 mice (Janvier, Le Genest-St Isle, France) at 3–4 months of age and ca. 23 g were anesthetized using isoflurane. Anesthesia was induced with 3.5% isoflurane and maintained at 1.5% in an oxygen/air (20%/80%) mixture. Mice were intubated and artificially ventilated (80 breaths per minute, bpm) using a small animal ventilator (CWE, Ardmore, USA) for the entire duration of the experiment. Stereotactic fixation was used to ensure reproducible positioning of the animal on the support. The body temperature was kept at 37 °C throughout the duration of the experiment. A catheter was placed in the tail vein for intravenous injection of pancuronium bromide (1 mg/kg; Sigma-Aldrich, Steinheim, Germany) as a bolus in order to avoid movement during acquisition and stimulation, respectively. For the electrical stimulation, a pair of bipolar platinum needle electrodes (Genuine Grass Instruments, West Warwick, USA) was inserted subcutaneously into the right hindpaw with a distance of 2 mm between the two needles. The numbers of animals for the different stimulus amplitudes and MRI readouts, respectively, are indicated in the figure legends.

### Electrical stimulation

The stimulation protocol has been described in detail earlier^4^. In brief, a pair of needle electrodes placed under the skin of the right hindpaw was connected to a current stimulus isolator (A365D, World Precision Instruments Inc., Sarasota, USA). The stimulus delivery was controlled with custom-written LabVIEW software (National Instruments, Austin, USA) and synchronized to the onset of the functional MRS and fMRI imaging sequence by a trigger signal from the MR scanner. A fixed set of stimulation parameters (current amplitude = 1 mA, 2 mA, or 3 mA, pulse duration = 0.5 ms, pulse frequency = 5 Hz) was used in all experiments. Generally, the stimulation paradigm consisted of ten cycles of 40 s stimulus period and 20 s post-stimulus period.

### MRI hardware

All experiments were carried out using a BioSpec 94/30 (Bruker BioSpin MRI GmbH, Ettlingen, Germany) small animal MR system operating at 400 MHz and equipped with a BGA 12AS HP gradient system with a maximum gradient strength of 400 mT/m and minimum rise time of 70 μs. The shim system allows shimming up to the second order. A four-element receive-only cryogenic phased array surface coil (2 × 2 geometry, overall coil size 20 × 27 mm^2^) with the coil system operating at 30 K (Bruker BioSpin AG, Fällanden, Switzerland) was used in combination with a circularly polarized 86 mm volume resonator for transmission.

### Spectroscopic imaging, MRSI

The protocols for anatomical and spectroscopic imaging have been described earlier^23^. Prior to SI, anatomical images were acquired for accurate positioning of the MRSI slice and the volume of interest. T2-weighted images (RARE sequence with repetition time/echo time (TR/TE) = 3500/14 ms, 66 × 66 μm^2^, slice thickness = 0.5 mm) were acquired in coronal and horizontal directions, followed by a reference image corresponding to the MRSI slice geometry. A coronal slice spanning a region from Bregma +1.34 mm to Bregma +0.02 mm was selected according to a stereotaxic mouse brain atlas^25^ for high resolution MRSI of a cortical region-of-interest (ROI) comprising 4 to 5 voxels. The total volume of interest was composed of 20 voxels of 1 µl. MRSI was performed using FIDLOVS^24^. First and second order shim gradients were adjusted in the volume of interest using previously acquired field maps. Prior to excitation, water suppression (WS) pulses^26^ were applied interleaved with 7 saturation pulses delimiting the volume of interest. Efficient saturation was achieved using hyperbolic secant shaped pulses with phase and amplitude modulation of the B_1_ field, applied with a bandwidth of 20 kHz. After WS and outer volume suppression (OVS), a frequency selective 90° sinc3 pulse was applied (bandwidth of 6 kHz) in combination with a slice selective gradient for excitation of a slice of 1.37 mm nominal thickness. The resulting FID signals were collected after an acquisition delay of 1.29 ms, TR = 2500 ms, spectral width = 8 kHz with 4096 data points (spectral resolution = 2 Hz/point). Parameter settings for the coronal slice were: Field of view = 15 × 15 mm^2^, Matrix size = 17 × 17, resulting in the following spatial resolution: 0.88 × 0.88 × 1.37 = 1 μl (the k-space was weighted with a Hanning function), and 12 min acquisition time. For each animal, the MRSI acquisition was repeatedly applied: three times for baseline measurements (pre-1 to pre-3), once during electrical hindpaw stimulation (stim), and three times after stimulation (post-1 to post-3), i.e. in total 7 acquisitions spanning a total of 84 min.

### fMRI

Twelve adjacent coronal slices of 0.5 mm slice thickness spanning all the regions covered in the MRSI experiments have been recorded. The first slice was positioned at Bregma +2 mm according to a stereotaxic mouse brain atlas^25^. The local field homogeneity was optimized in the area of interest using field maps. BOLD fMRI data prior and during electrical stimulation were acquired using a gradient-echo echo-planar imaging (GE-EPI) sequence with the following parameters: Field of view = 16 × 7 mm^2^, Matrix size = 80 × 35, yielding an in-plane voxel dimension of 200 × 200 µm, TR/TE = 1000/12 ms, Averages = 1, yielding a temporal resolution of 1 s, with interleaved acquisition of slices. A data set comprised 2160 repetitions; resulting in a total acquisition time of 36 min. Electrical stimulation (ten stimulation cycles of 40 s stimulus period and 20 s post-stimulus period) was started after recording baseline data for 12 min.

### Data analysis

Data analysis was performed in an automated fashion using LC model^27^. Spectral processing includes zero and first order phase correction, windowing (last 500 points of the FID set to zero) and filtering of the remaining water signal. The strong first order phase shift introduced by the time delay between excitation pulse and FID acquisition (1.29 ms) was compensated by a shift in the time domain and considering the maximal phase difference of 360 degrees for each dwell time. No further pre-processing was applied to the data prior to quantification. Relative quantification was performed using total creatine (PCr + Cr) as an internal reference. Statistical analysis was performed in R 3.2.4 (The R Foundation for Statistical Computing, Vienna, Austria). For each animal, a baseline value was calculated from the average of the three pre-stimulation MRSI scans. Comparison between baseline and stimulus period for the three stimulus amplitudes was performed using a linear mixed model from lme4 package, with metabolite concentration as response variable, stimulus amplitude together with condition (baseline or stimulation) as fixed effect, and individual animal intercepts as random effect. In order to account for potential inaccuracies in the estimation of some metabolite concentrations, CRLB values were used as weighting factors in the fitting process. The assumption of normality of the residuals was tested with QQ-plots to inspect normal distribution, Tukey–Anscombe plots for the homogeneity of the variance and skewness, and scale location plots for homoscedasticity. Normality of residual was considered plausible. Pairwise comparison between baseline and stimulation for each of the three amplitudes was performed using general linear hypothesis tests (LH-test) with multcomp package. To determine the presence of an incremental effect with increasing stimulation strength, percentage metabolite concentration change relative to baseline were used as a response variable in a linear model, and stimulus amplitudes, including control stimulation at 0 mA (needles are placed but no stimulation is applied), was modelled as explanatory variable in a linear model. An analysis of variance (ANOVA) was used to determine the presence of an amplitude effect. Post-hoc pairwise comparisons between amplitudes were carried using two-sample t-test. The null hypothesis, i.e., the absence of a pairwise difference between stimulation and baseline, or amplitude effect, was rejected at p ≤ 0.05, uncorrected.

In order to visually compare spectra acquired at rest and during stimulation (Fig. 2), a general line broadening was applied to the latter to match the NAA linewidth of the two spectra. For quantitative analysis, uncorrected spectra have been used. We observed a decrease in spectral resolution with increasing stimulus amplitude as reflected by the separation of the PCr/Cr resonances at 3.9 and the taurine (Tau)/(PCr + Cr) signal at 3.2 ppm.

FMRI preprocessing was performed in AFNI (http://afni.nimh.nih.gov) following previously published procedures^28^. The first 40 repetitions were dismissed to account for the T1 relaxation effect. Data were slice-time corrected. Motion correction was performed by realigning all scan volumes to the first repetition and coregistration of all animal brains to a strain-specific template. Further, a weak Gaussian blur (FWHM = 0.3 mm) was applied. Data were scaled to percent signal change relative to baseline and detrended by adding polynomials (to the fourth order) as regressors to a general linear model (GLM). The statistical parametric maps were generated using the GLM. As a model for the evoked BOLD response upon electrical hindpaw stimulation, the SPM basis functions were convolved with the stimulus time course. The beta values of the GLM analysis using the gamma variate as a regressor entered the group statistics (one-sample t-test). Maps are shown as color-coded beta-values. For time course analysis of fMRI data, MATLAB based software AFNI was used to define ROIs according to a stereotaxic mouse brain atlas^25^ for the contralateral and ipsilateral primary somatosensory hindlimb cortex (S1HL). Signal changes are expressed as percentages relative to baseline starting from repetition 40. Maximum BOLD signal change and the integral were quantified for the activation period upto 720 s after stimulus onset, and the Pearson correlation between contra- and ipsilateral S1HL was computed.

## Results

### BOLD fMRI response upon unilateral paw stimulation

**Figure 1:**
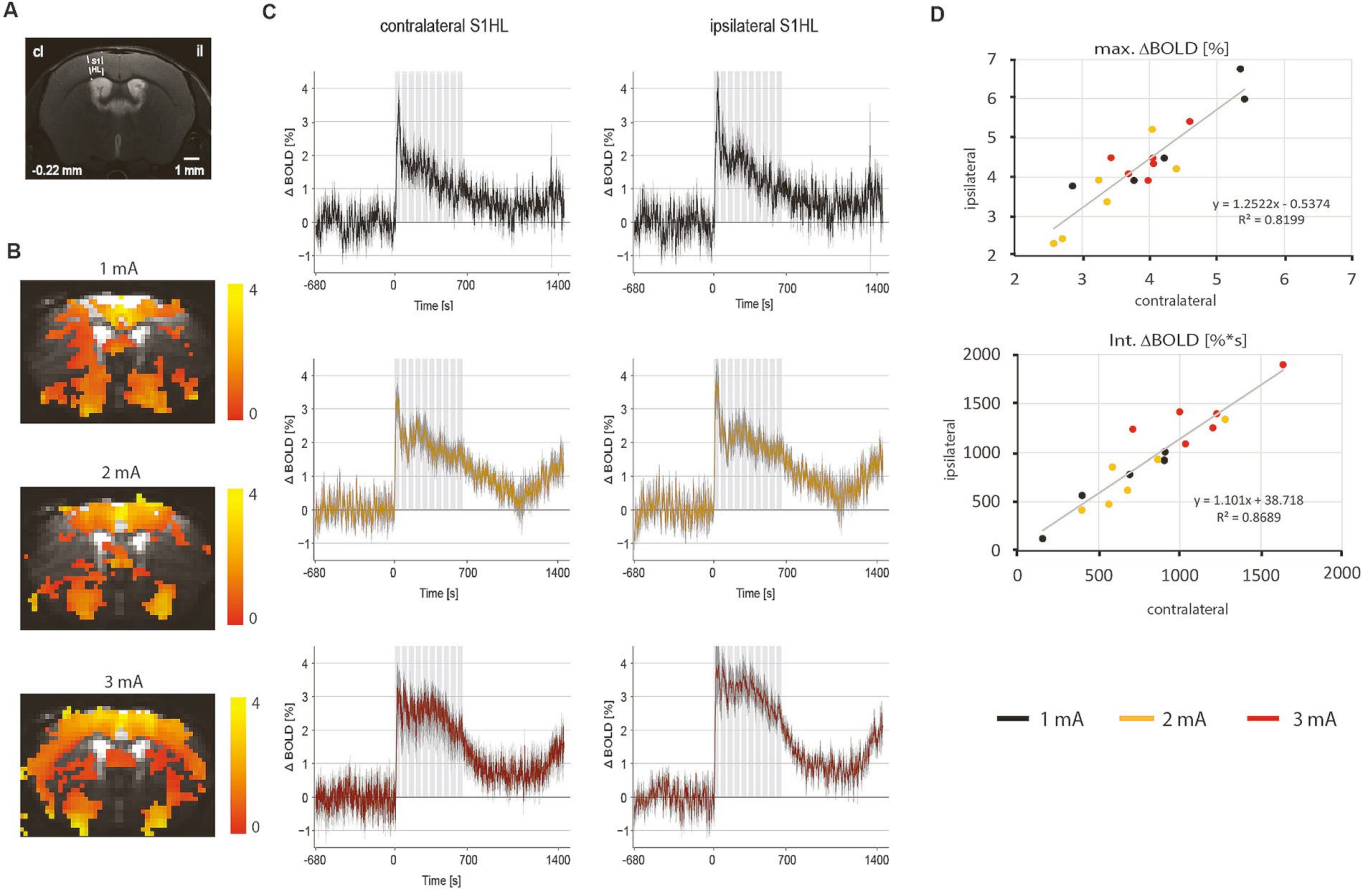
(**A**) T_2_-weighted anatomical image with distance to Bregma and primary somatosensory. Hindlimb cortex contralateral to the stimulated paw (S1HL) indicated. (**B**) Statistical parametric maps of BOLD signal changes (ΔBOLD) elicited by electrical hindpaw stimulation at 1, 2, and 3 mA for N = 5 animals (for 1 mA) and N = 6 animals (for 2 mA and 3 mA). Maps are shown as color-coded beta-values. (**C**) Average signal time courses per stimulation amplitude show stimulus-evoked ΔBOLD extracted from the contralateral and ipsilateral S1HL (mean +− standard deviation). Note that contra- and ipsilateral signal changes look almost identical. The gray rectangle indicates the stimulation period after a baseline period of 720 repetitions (s). (**D**) Correlation values R^2^ between contra- and ipsilateral responses are given in the plots for the maximum ΔBOLD amplitude values and for the integral of the contra- and ipsilateral stimulus-evoked signal changes in the first 720 repetitions (s) after stimulus onset. Different current amplitudes are indicated by colours. Data points represent individual animals.

Mean BOLD signal changes in percent of baseline values (ΔBOLD) elicited by electrical hindpaw stimulation and statistical parametric maps are shown for stimulus amplitudes of 1 mA, 2 mA, and 3 mA, respectively (Fig. 1). Time courses were extracted from contralateral and ipsilateral S1HL. In all cases the BOLD signal displayed an ‘initial peak’ of approximately 60 s duration upon onset of stimulation that could not be sustained but decreased slowly for 1 mA and 2 mA or leveled off to a ‘steady state’ value for 3 mA for the rest of the stimulus period. Signal increases returned to baseline only slowly after the last of the ten stimulation blocks.

Statistical parametric maps displaying brain activation indicated that BOLD responses were not confined to contralateral S1HL, thalamus, and secondary somatosensory cortex (S2), irrespective of the stimulus strength applied, but showed a rather bilateral and widespread pattern. The average positive BOLD responses calculated from values between onset of the first stimulus period and return to baseline level in the analyzed regions ipsilateral to stimulation were almost identical to those in the corresponding contralateral ROIs (Fig. 1) in line with previous observations^4,29,30^. While the integral values were found to depend on the stimulus amplitude, whereas this was not the case for the maximum peak amplitude.

### Functional MRSI response upon unilateral paw stimulation

Metabolite maps were acquired with a nominal spatial resolution of 1 μl and a temporal resolution of 12 min before, during and after electrical stimulation of the right hindpaw.

**Figure 2:**
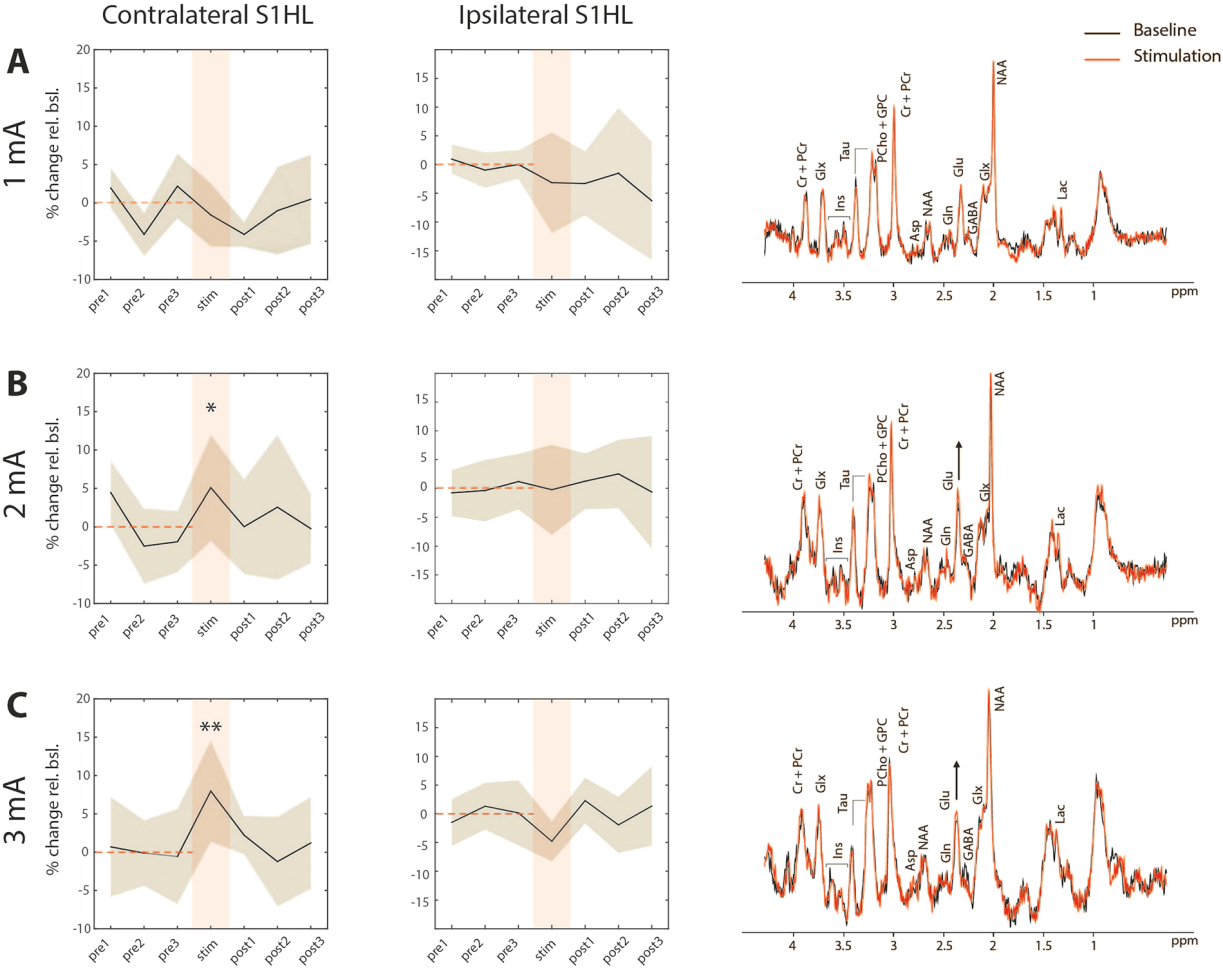
Glu time courses and representative spectra extracted from the contralateral (left) and ipsilateral (right) S1HL before (black) and during (red) electrical hindpaw stimulation with 1 mA (**A**, N = 6), 2 mA (**B**, N = 8) and 3 mA (**C**, N = 6). Glu levels were significantly increased when stimulating with 2 and 3 mA (*p ≤ 0.05, **p ≤ 0.01). For quantitative and statistical analysis, an average value of the pre-stimulation values (indicated by the red dashed line) was calculated and used as baseline value. The shaded area represents standard deviation. Representative pre- (black line) and post-stimulation (red line) spectra for all stimulus amplitudes are overlayed. Labels indicate resonances of phosphocreatine (PCr), creatine (Cr), glutamate/glutamine (Glx), glutamate (Glu), glutamine (Gln), inositol (Ins), taurine (Tau), phosphorylcholine (PCho), glycero-phosphorylcholine (GPC), aspartate (Asp), N-acetyl-aspartate (NAA), γ-butyric acid (GABA), and lactate (Lac). Post-processing of spectra included phase correction (zero and first order), filtering of the remaining water signal and windowing. Line broadening was applied to match the line width of the NAA signal between pre- and post-stimulation spectra to compensate for the BOLD effect.

Highly resolved spectra extracted from single voxel located in the contralateral and ipsilateral S1HL allowed assigning the resonances of at least 10 metabolites, including the Glu signal at 2.35 ppm (Fig. 2). The Cramer Rao Lower Band (CRLB) for Glu estimated using LCmodel were 3% on average throughout the experiment, 10% for Gln, 10 to 15% for GABA, and 15–20% for Lac (Suppl. Fig. 1). Results of the quantitative analysis of the Glu signal are shown in Fig. 2. While no significant Glu change was measured in the 1 μl voxel located in contralateral S1HL when stimulating with 1 mA, individual time courses indicated elevated Glu levels relative to baseline for stimulus amplitudes 2 mA and 3 mA, with Glu increases of 5% ± 5 and 8% ± 7, respectively. None of the other metabolites analyzed displayed significant changes upon electrical hindpaw stimulation except for Lac (Suppl. Fig. 2).

**Figure 3:**
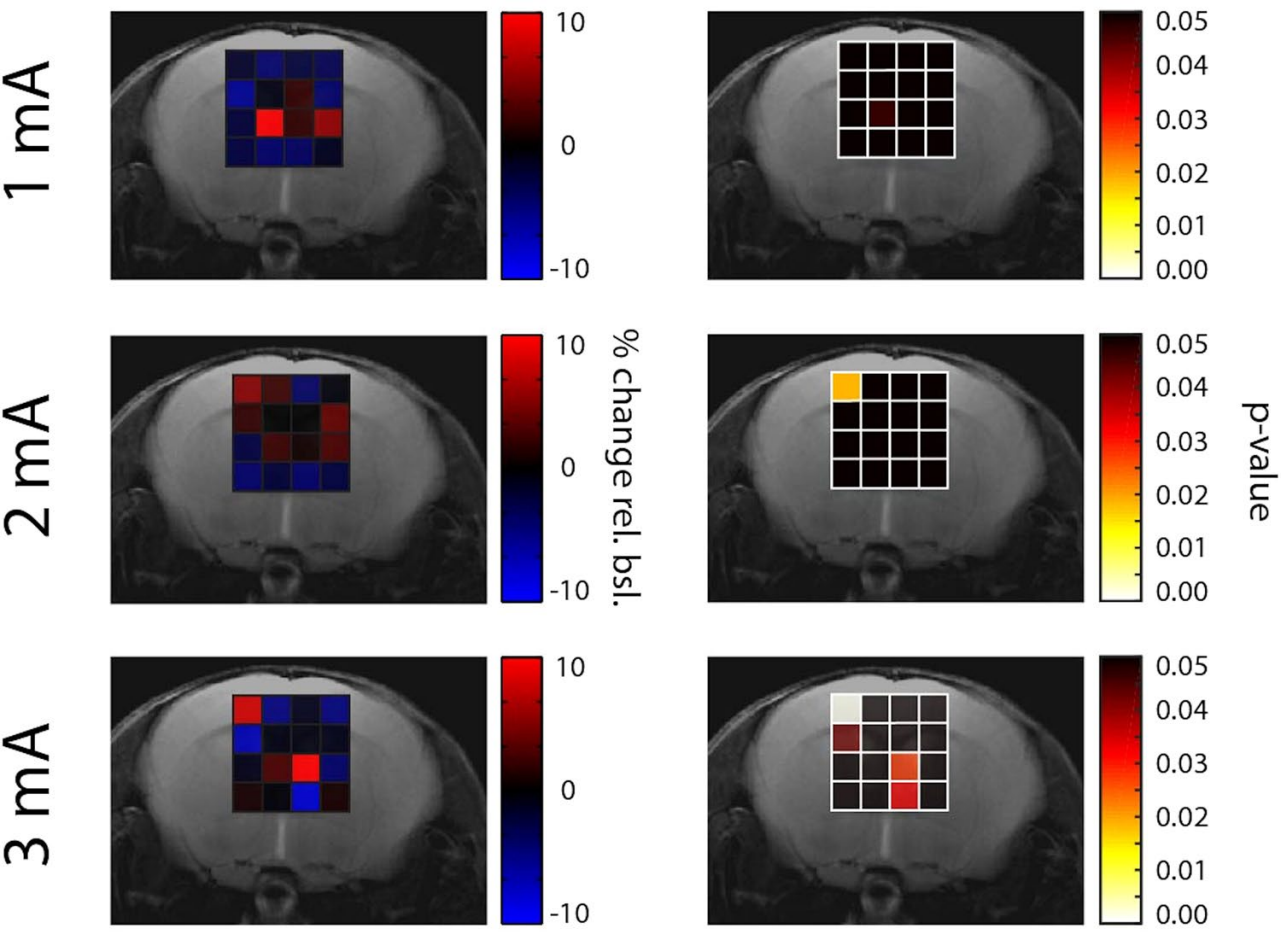
Maps of mean percentage change and statistical maps illustrating the spatial specificity of the Glu response elicited by unilateral electrical hindpaw stimulation with 1, 2 and 3 mA (from same animals plotted in Fig. 2). The mean Glu maps of animals stimulated with 2 mA and 3 mA display the highest response in the contralateral (left) S1HL, indicating regional specificity. This result is confirmed by the statistical analysis showing distinct significant voxels in the contralateral S1HL.

**Figure 4:**
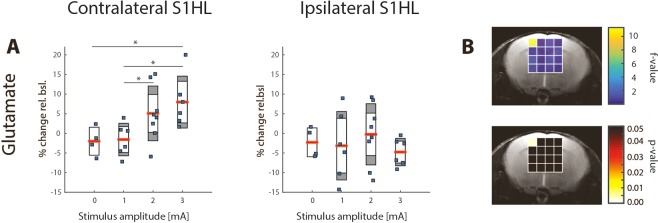
Glu response in the contra- (left) and ipsilateral (right) cortical S1HL area as a function of the stimulus amplitudes 0 mA (N = 4), 1, 2 and 3 mA (from same animals plotted in Fig. 2). The Glu response in contralateral S1HL exhibits a positive correlation with the stimulus amplitude while this not was not the case for the Glu signal in ipsilateral S1HL, which at no current amplitude displayed values different from baseline. A statistical test probing for a linear dose-dependence of Glu levels in function of stimulation amplitude revealed only one significant voxel located in the contralateral S1HL (left) in the Glu map.

Glu changes triggered by stimulation with 2 mA and 3 mA were found to be higher in the primary somatosensory cortex contralateral to the stimulated paw (contralateral S1HL) as compared to all other analyzed cortical voxels in the FOV, and in particular to the corresponding cortical region ipsilateral to the stimulated paw (Figs 3 and 4). Changes for the respective stimulus amplitudes in the contralateral hemisphere were: −2.5% ± 2.5 for 0 mA, −2.3% ± 2.6 for 1 mA, 6.2% ± 7.5 for 2 mA, and 8.1% ± 6.9 for 3 mA. In contrast, changes in the ipsilateral hemisphere were smaller particularly for 2 mA and 3 mA: −2.5% ± 2.5 for 0 mA, −3.2% ± 8.4 for 1 mA, −0.9% ± 7.8 for 2 mA, and −4.9% ± 4.5 for 3 mA. The spatial specificity of the Glu response was also demonstrated in statistical maps for stimulus amplitudes of 2 mA and 3 mA, which revealed significant signal changes in the contralateral (p ≤ 0.02 and ≤0.01 for 2 mA and 3 mA, respectively) but not in the ipsilateral S1 region. No voxel displaying significant changes in Glu intensity has been observed for a stimulus amplitude of 1 mA. A statistical test probing for a linear dependence of Glu signals on the current amplitude was performed in all voxels (Fig. 4). There was only one 1 μl voxel located in the contralateral S1HL displaying a highly significant linear dependence of Glu signals on the stimulus amplitude (p ≤ 0.01), while for all other voxels p > 0.1.

## Discussion

Neural activity is associated with increased neurotransmitter turnover and energy demand, i.e. adaptations in metabolic activity that are matched by local regulation of cerebral blood flow (CBF) and oxygen supply. A result of neurovascular coupling balancing oxygen delivery to and oxygen consumption by the tissue is the generation of BOLD contrast. Any fluctuation affecting CBF or arterial oxygen saturation might alter oxygen supply and hence generate BOLD responses that are not associated with neural activity. In fact, it has been reported that in mice stimulus-evoked BOLD responses appear to be dominated by general arousal-associated neural activity- and cardiovascular-related hemodynamic alterations^4^. Thus, readouts of brain function that are not based on cerebral hemodynamics become attractive. The intimate link of neurotransmitter turnover and energy metabolism to neural activity renders monitoring of stimulus-induced adaptations in cerebral metabolism in a spatially resolved manner across extended brain areas an attractive alternative for functional brain studies complementing BOLD fMRI, though the inferior sensitivity of MRSI constitutes a considerable limitation.

A major challenge when applying time-resolved MRSI relates to limited temporal resolution due to intrinsic poor sensitivity and the sequential nature of image acquisition. This issue was addressed by combining the high sensitivity provided by a cryogenic phased array receive-only coil with a MRSI method based on FID acquisition with short acquisition delay, yielding nearly full signal intensity. In a previous study we have demonstrated feasibility of this approach for monitoring metabolic changes in mouse brain upon administration of the GABA_A_ receptor antagonist bicuculline. This paradigm is known to elicit a strong widespread excitatory response, which may lead to dysregulation of energy metabolism^20^ and failure of neurovascular regulation^23,31^. The results of the current study demonstrate that the method provides sufficient sensitivity to quantitatively assess Glu changes in response to a more physiological sensory stimulation paradigm triggering discrete and localized metabolic events. The MRSI readout was of sufficient sensitivity to quantitatively assess changes in Glu levels in the mouse brain, the amplitude of which depended on the current amplitude of the electrical stimulus that was unilaterally applied to the hindpaw. Changes in Glu signal amplitude of the order of 5 to 8% could be reliably measured for stimulus amplitudes of 2 mA and 3 mA (p≤0.02 and ≤0.01, respectively).

The major result of this study is that in isoflurane-anaesthetized mice the changes in the Glu signal upon unilateral sensory stimulation appeared confined to the contralateral S1HL cortical area. No other voxel within the transverse MRSI data set comprising cortical and striatal structures displayed consistent changes in the Glu signal amplitude with respect to the increasing intensity of the stimulus amplitude. This holds in particular for the Glu signal in ipsilateral S1HL, which did not display any dependence on the stimulus. While stimulus-evoked changes in Glu signal intensity clearly indicated region-specific processing of peripheral input, this was not the case for the BOLD fMRI readout, which showed the widespread bilateral response pattern reported earlier^4^. Hence, metabolic readouts constitutes an attractive complementation to fMRI, while MRSI approaches, enabling the study of metabolic adaptations across extended brain areas, are essential for such studies as they allow analyzing region-specific responses simultaneously, i.e. to the identical stimulus.

As no comparable studies have been carried out in mice to date we compared the extent of Glu changes in response to hindpaw stimulation to ^1^H-MRS data obtained from rats and humans reporting changes in Glu levels upon sensory stimulation. Recently, a raise in Glu levels by approximately 8% in response to electrical stimulation of the trigeminal nerves in rats was reported using SVS^16^. As no changes in the Gln signal we observed, Glu increases were attributed to an increased flux through the malate-aspartate shuttle as corresponding decreases in aspartate (Asp) have been measured. In humans, similar observations were made upon visual stimulation^9^ but not upon motor activation^18^. In our study we did not observe statistically significant changes in Asp levels (Suppl. Fig. 2); the CRLB of Asp were found to be 35% on average representing a too large uncertainty compared to the effect size expected. Nevertheless, the Glu-related observations described by Just *et al*.^16^ are in line with our results. In contrast, a study of Xu *et al*.^17^ involving forepaw electrical stimulation in rats reported the opposite: a decrease in Glu and a corresponding increase in Gln was measured, both attributed to Glu-Gln cycling. The PCr/Cr ratio was found decreased, while no changes in Lac levels were measured. Similar to Just *et al*.^16^, we did not observe a significant decrease in the Gln signal (though there was a tendency of a decrease, which, however, did not reach statistical significance; Suppl. Fig. 2) in line with the increase in the Glu signal probably due to lack of sensitivity as reflected by the CRLB for Gln of 9.5%. Hence, it remains unclear whether the increased Glu levels reflect increased flux through the malate aspartate-shuttle or increased Glu-Gln cycling.

In our analysis, we focused on changes in Glu levels as we searched for a readout that is more intimately linked to neural activity than the hemodynamic BOLD response. This is also the case for metabolites associated to energy metabolism such as Lac, for which significant changes have been found, despite relative high values for the CRLB. Nevertheless, these signal changes turned out to be less specific and rather mimic the widespread BOLD response. We cannot conclude at this stage whether this result is specific for our study or whether it reflects a general feature as most studies published reporting stimulus-induced increase of lactate used SVS and did not investigated apparently not affected brain regions nor did they analyze the Lac response as a function of the stimulus intensity^8–10,16–18,21,22,32^.

The major limitations of MRS and MRSI as brain activity readout is sensitivity. As expected, the small voxel volumes (1 µl) and high demands on temporal resolution in mouse MRSI rendered accurate quantification of changes in metabolite concentrations in the mouse brain a challenging task. The CRLB for Glu quantification were found to be 3% on average, which implies that with a groups size of N = 6, an effect size of >5% can be reliably detected, which was the case for stimulus amplitudes of 2 mA and higher. For an extrapolated effect of 2 to 3% at 1 mA, the corresponding group size would amount to >15 mice. Apart from intrinsic biological variability, MRSI methodological aspects might contribute to uncertainties in concentration estimates. Spectra may be contaminated by lipid signals arising from imperfect volume selection or by residual water signal. Moreover, the short acquisition delay associated with FID sampling comes at the expense of significant signal contribution arising from macromolecules, which may compromise baseline estimation and hence quantification accuracy. The longitudinal design of the study, i.e. assessing concentration changes with regard to the baseline condition may account for this effect to some extent, as we may assume this confounds to remain constant throughout the experiment.

In conclusion, the MRSI approach used, in contrast to SVS, allowed monitoring changes in cerebral Glu level associated with neural activity elicited by a sensory stimulus in mice in a spatially resolved manner, providing information on active and non-active regions simultaneously, a prerequisite for analyzing the specificity of induced changes in neurotransmitter and metabolite levels. A major motivation for the current study was our intriguing observation of widespread BOLD fMRI responses to unilateral hindpaw stimulation in isoflurane-anaesthetized mice involving large cortical and subcortical domains in both hemispheres^4^. The same paradigm led to dose-dependent increases in the Glu signal in S1HL cortical area contralateral to the stimulation, but not in the respective ipsilateral territory demonstrating regional specificity of the non-hemodynamic readout. Limitations of functional MRS/SI are low nominal spatial (∼1 μl) and temporal resolution (∼10 min), preventing the analysis of dynamic changes observed in the BOLD response. Also the use of moderate to strong stimuli of rather long duration is required. Nevertheless, Glu mapping using MRSI could become an interesting complement to BOLD fMRI in mice, in particular when applying strong sensory/noxious stimuli, for which severe contamination of neurogenic BOLD signals by systemic responses would be expected.

## Supplementary information

Supplementary material

## Data Availability

Data generated during the study will be available in a public repository.
