# Supplementary material for: Functional spectroscopic imaging reveals specificity of glutamate response in mouse brain to peripheral sensory stimulation
Source: Sci Rep. 2019 Jul 22;9:10563. doi: 10.1038/s41598-019-46477-1 (PMC6646328; doi:10.1038/s41598-019-46477-1)
Supplement: Supplementary file 1 — Supplementary material [file 41598_2019_46477_MOESM1_ESM.pdf]

Title:

Functional spectroscopic imaging reveals specificity of glutamate response in mouse brain to peripheral sensory stimulation

Authors:

Aline Seuwen[1,2 *], Aileen Schroeter[1,2 *], Joanes Grandjean[1,3,], Felix Schlegel[1], Markus Rudin[1,2,4]

* These authors contributed equally to this work.

Author's affiliation:

[1] Institute for Biomedical Engineering, University and ETH Zurich, Wolfgang-Pauli-Str. 27, 8093 Zurich, Switzerland

[2] Neuroscience Center Zurich, University and ETH Zurich, Winterthurer-Str. 190, 8057 Zurich, Switzerland

[3] Singapore BioImaging Consortium, Agency for Science, Technology, and Research, 11 Biopolis way, Singapore 138667, Singapore

[4] Institute of Pharmacology and Toxicology, University of Zurich, Winterthurer-Str. 190, 8057 Zurich, Switzerland

Corresponding author:

Markus Rudin, ETH Zurich, Institute for Biomedical Engineering, Wolfgang-Pauli-Str. 27, 8093 Zurich, Switzerland

Phone: +41 44 633 76 04, E-Mail: rudin@biomed.ee.ethz.ch

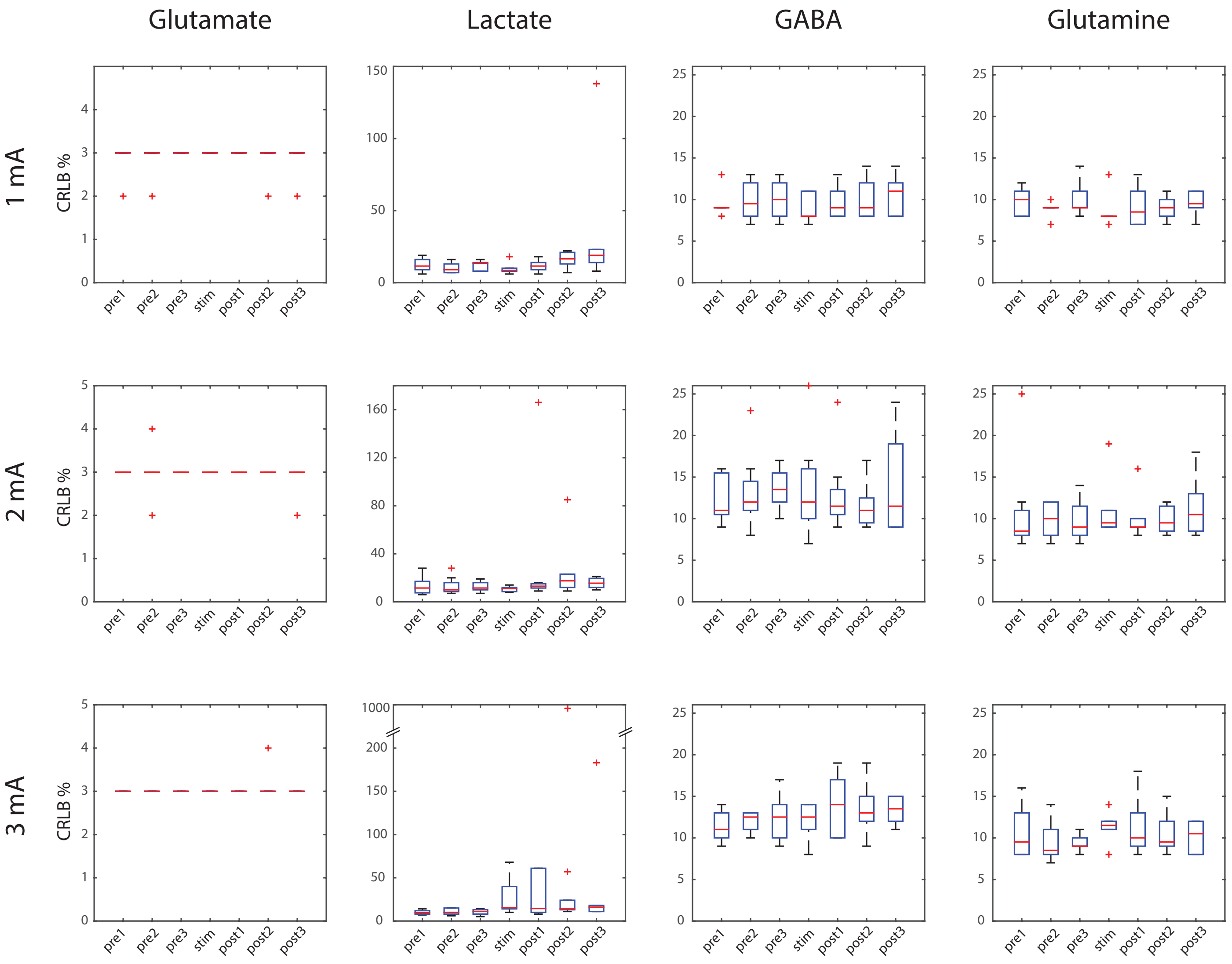

Glutamate
Lactate
GABA
Glutamine
1 mA
2 mA
3 mA
CRLB %
pre1
pre2
pre3
stim
post1
post2
post3

Suppl. Fig. 1: CRLB for Glu, Lac, GABA and Gln as extracted from the contralateral S1HL area plotted for every time point. CRLB did not change significantly in the course of the experiment, indicating that the data quality and the reliability of quantification was maintained over time and despite the sensory stimulus. Variability due to movement is not expected since animals were paralysed and mechanically ventilated.

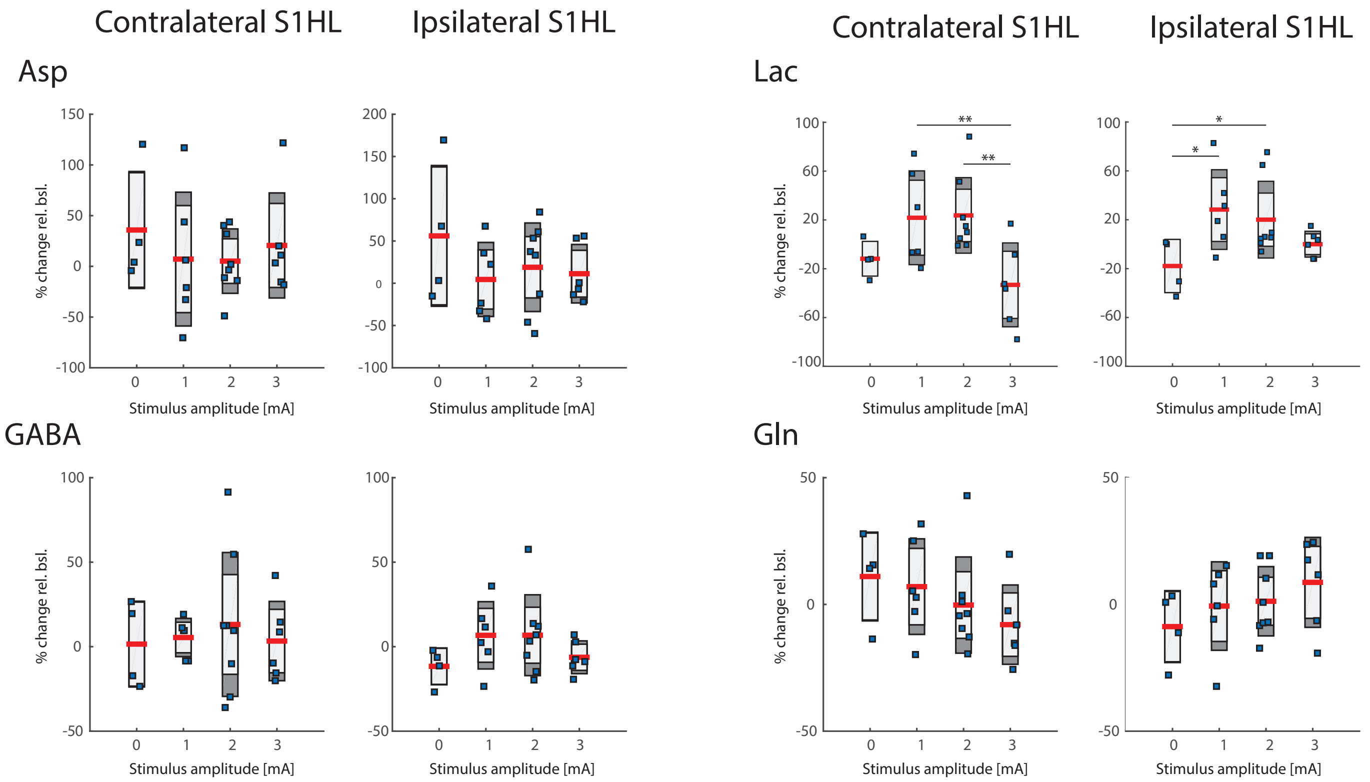

Contralateral S1HL
Ipsilateral S1HL
Contralateral S1HL
Ipsilateral S1HL
Asp
Lac
GABA
Gln
% change rel. bsl.
Stimulus amplitude [mA]
0
1
2
3
150
100
50
0
-50
-100
200
60
20
-20
-60
*
**

Suppl. Fig. 2: Changes in Asp, Lac, GABA, and Gln, in percent of baseline values measured in the contralateral and ipsilateral S1HL region for the stimulus amplitudes 0, 1, 2 and 3 mA (from same animals plotted in Figs. 2,3,4). No significant change has been found except for Lac. Gln values tended to decrease with increasing stimulation amplitude in the contralateral S1HL region though changes did not reach significance.
